# Potential Distribution Predicted for *Rhynchophorus ferrugineus* in China under Different Climate Warming Scenarios

**DOI:** 10.1371/journal.pone.0141111

**Published:** 2015-10-23

**Authors:** Xuezhen Ge, Shanyong He, Tao Wang, Wei Yan, Shixiang Zong

**Affiliations:** 1 Key Laboratory of Beijing for the Control of Forest Pests, Beijing Forestry University, Beijing, China; 2 Mentougou Forestry Station, Beijing, China; 3 Coconut Research Institute, Chinese Academy of Tropical Agricultural Sciences, Wenchang, Hainan, China; Institute of Zoology, CHINA

## Abstract

As the primary pest of palm trees, *Rhynchophorus ferrugineus* (Olivier) (Coleoptera: Curculionidae) has caused serious harm to palms since it first invaded China. The present study used CLIMEX 1.1 to predict the potential distribution of *R*. *ferrugineus* in China according to both current climate data (1981–2010) and future climate warming estimates based on simulated climate data for the 2020s (2011–2040) provided by the Tyndall Center for Climate Change Research (TYN SC 2.0). Additionally, the Ecoclimatic Index (EI) values calculated for different climatic conditions (current and future, as simulated by the B2 scenario) were compared. Areas with a suitable climate for *R*. *ferrugineus* distribution were located primarily in central China according to the current climate data, with the northern boundary of the distribution reaching to 40.1°N and including Tibet, north Sichuan, central Shaanxi, south Shanxi, and east Hebei. There was little difference in the potential distribution predicted by the four emission scenarios according to future climate warming estimates. The primary prediction under future climate warming models was that, compared with the current climate model, the number of highly favorable habitats would increase significantly and expand into northern China, whereas the number of both favorable and marginally favorable habitats would decrease. Contrast analysis of EI values suggested that climate change and the density of site distribution were the main effectors of the changes in EI values. These results will help to improve control measures, prevent the spread of this pest, and revise the targeted quarantine areas.

## Introduction

The red palm weevil, *Rhynchophorus ferrugineus* (Olivier) (Coleoptera: Curculionidae) is the primary pest of palms (Arecaceae) and is included on the A2 list of pests recommended for regulation (this list includes pests present in part of EPPO region, but not widely distributed within it, that are officially controlled) [1]. The weevil is native to southern Asia and Melanesia and has a wide geographical distribution, including Oceania, Asia, Africa, America, and the EPPO region [2]. In China, *R*. *ferrugineus* was first reported in Zhongshan City, Guangdong Province, in 1997 [3]. The pest then spread widely into many areas, particularly into southern provinces such as Hainan, Guangxi, and Yunnan [4]. In China, hosts susceptible to *R*. *ferrugineus* were initially identified by Feng and Liu [5]; their findings were based on reports in the literature and expert opinion. The identified hosts are *Cocos nucifera* L., *Phoenix dactylif*era L., *Metroxylon sagu* Rottb., *Arenga pinnata* (Wurmb.) Merr., *Elaeis guineensis* Jacq., *Corypha gebanga* Bl., *Phoenix sylvestris* Roxb., *Borassus flabellifer* L., *Caryota maxoma* Blume., *C*. *cumingii* Lodd., *Oreodox aregia* HBK., and *Areca catechu* L. Of these, *C*. *nucifera* L. and *P*. *dactylifera* L sustain most damage.

Since the *R*. *ferrugineus* invasion of China, large numbers of coconut trees were harmed in multiple locations in Hainan Province; the area of damage measured nearly 10,000 km^2^, and almost 20,000 coconut trees were killed. Furthermore, palms were seriously damaged in southern China, and the area of damage is rapidly expanding. The outbreaks of *R*. *ferrugineus* not only seriously affected the development of the coconut and areca nut industries, but also caused a huge threat to the green ecological security of the coastal area of China [6–8]. Thus, measures to control the outbreak of *R*. *ferrugineus* must be taken to protect the environment and to reduce economic loss.

Different control methods to manage *R*. *ferrugineus* in China include survey and removal of heavily infested trees, pheromone traps, application of chemical insecticides, and use of entomopathogenic nematodes as a form of biological control [9]. Related technologies to control the spread and outbreaks have matured; however, preventive measures are not completely effective. Identifying the potential distribution of *R*. *ferrugineus* would help to focus effective inspection and quarantine protocols to prevent the continued dissemination of the pest. A relative measure of the likely performance of the pest in a given location has implications for local management.

The main factor that influences climate change is human activity. The pace of development in different countries leads to different greenhouse gas emission scenarios, all of which influence the climate. The international community has no fully recognized the reality of global warming. The international community recently recognized global climate warming by The Intergovernmental Panel on Climate Change (IPCC) proposed that average global temperatures are projected to increase by 1.8–4°C by the end of the 21^st^ century under several greenhouse gas emission scenarios [10]. Climate change will profoundly affect the distribution and abundance of all species, including insects, and promote ecology-driven changes in phenology [11]. Climate change may result in potential expansion of insect populations to polar regions and higher elevations [12]. Increases in global temperatures will expand or reduce the range of insects by converting climatically unsuitable habitats into suitable ones or vice versa [13]. Therefore, identifying the potential distribution of *R*. *ferrugineus* under different global warming scenarios is crucial if we are to develop effective prevention and control measures.

Species distribution models are used to predict the suitability of the climate for a particular species. These bioclimatic models include ANUCLIM/BIOCLIM, CLIMATE, CLIMEX, DOMAIN, GARP, HABITAT, and MaxEnt [14]. CLIMEX, GARP, HABITAT, and MaxEnt are popular tools, which are widely used in estimate the potential distribution of species. However, future climate data is difficult to collect and sort in accordance with the requirements of GARP, HABITAT, and MaxEnt, as these models do not take the biological characteristics of species into account. Therefore, CLIMEX is most often used to forecast the potential distribution of different insects, including *Bactrocera dorsalis* (Hendel) and *Hemiberlesia rapax* (Comstock), in different countries under different global climate change scenarios [15–23]. The CLIMEX model is a dynamic simulation model, and one widely used function is ‘Compare Locations’, which requires the user to input detailed biological information and the geographical distribution of pests.

Previous studies have examined the potential distribution of *R*. *ferrugineus*. Li et al. [24] and Ju et al [25] used the GARP model and the ‘Match Climates’ function of the CLIMEX model, respectively, to forecast potential distribution according to current climate data. Ju et al. [26] tentatively inferred the northern limit for overwintering *R*. *ferrugineus* according to cold hardiness and host distribution. Feng and Liu [5] used the MaxEnt model to predict the potential distribution of *R*. *ferrugineus* in China, and also considered host distribution. Fiaboe et al. [27] predicted the potential worldwide distribution of *R*. *ferrugineus* using ecological niche modeling (GARP and MaxEnt), although they did not provide a detailed description of the distribution in China. However, no previous studies have investigated areas that might become suitable for *R*. *ferrugineus* under different global warming scenarios.

Therefore, the aim of the present study was to forecast the potential distribution of *R*. *ferrugineus* in areas of China with a suitable climate according to current climate and the future climate data. The predictions were based on current climate data and simulated future climate data provided by the Tyndall Center for Climate Change Research; the function ‘Compare Locations’ within CLIMEX was used to complete the study. The results may help to identify potential changes in those areas with a suitable climate for *R*. *ferrugineus*, thereby providing a theoretical reference and a practical guide to facilitate effective quarantine, prevention, and control measures.

## Materials and Methods

### Study Model and Software

#### The CLIMEX model

CLIMEX is a dynamic simulation model that estimates the potential geographical distribution and relative abundance of a species according to climate [26]. The model is currently used worldwide to predict the potential distribution of insects, weeds, and annelids. Here, CLIMEX 1.1 (Hearne Scientific Software, Melbourne, Australia) was used to estimate the climatic suitability of China for *R*. *ferrugineus*. The ‘Compare Locations’ function debugs the CLIMEX parameters until the prediction is consistent with the known distribution; this requires the input of detailed biological information and the geographical distribution of the pest. Based on the available biological information for *R*. *ferrugineus*, the ‘Compare Locations’ function was used in this study. When setting the model parameters, the growth indices, stress indices, and two limiting conditions (obligate diapause index and length of the growing season) were combined into an Ecoclimatic Index (EI), which describes the climatic suitability of a location for a species. The stress indices (SI) describe the probability that the species can survive in a poor environment and are related to factors that limit the geographical distribution. The growth indices (GI) describe the intrinsic rate of increase of a species under ideal conditions, which are related to seasonal activity patterns and relative abundance. The EI values ranged from 0 to100. A small EI value indicated that the probability was low for the species to survive in a particular location, and a large EI value indicated that the probability was high. An EI value close to 0 indicated that the location was not favorable for the long-term survival of a species, whereas an EI value of 100 was only achievable under constant and ideal conditions, such as those in an incubator [28]. The suitable degree for a species’ survival that an EI value stands varies from species to species according to their particular characteristics.

#### ArcGIS software

After the CLIMEX results were obtained, ArcMap 9.3 (Environmental Systems Research Institute, Redlands, CA, USA) was used to perform a layer analysis and generate layout distribution maps. The inverse distance-weighted (IDW) interpolation was used to provide more surface detail and the thematic mapping function was used to create maps showing different climates suitable for *R*. *ferrugineus* in China.

### Data Collection

#### Current Chinese climate data

The China Surface Climate Monthly Standard Values data set (1981–2010) was downloaded from the China meteorological data sharing service system (http://cdc.cma.gov.cn/). This data set contained the available data from 885 meteorological stations, and the format of the data was converted to satisfy the requirements of the CLIMEX model. The data were then imported into CLIMEX. However, the climate data for Taiwan and Hong Kong did not exist within this data set; thus, the two meteorological stations in Taiwan (Taipei and Hualien) and the one station in Hong Kong (Hong Kong) were added from the data set used in CLIMEX. The entire set of climate data from 888 meteorological stations representing all regions of China was used for the historical Chinese climate data analysis.

#### Future Chinese climate data for the 2020s (2011–2040)

Data download: The TYN SC2.0 data set provided by the Climatic Research Unit at the University of East Anglia (http://www.cru.uea.ac.uk/cru/data/hag/) was used to extract future climate data. These data had very high resolution (0.5° × 0.5°) and included 67,420 global land grid points. The simulated climate data were generated by five climate-coupling models [HadCM3 (UK), PCM (USA), CGCM2 (Canada), CSIRO2 (Australia), and ECHAM4 (Germany)] using four greenhouse gas emission scenarios (A1FI, A2, B1, and B2). The TYN SC2.0 data set simulated five climate data sets for 2001–2100, which included the average daily temperature, diurnal temperature range, precipitation, vapor pressure, and cloudiness [29].

Selection of climate models, simulation duration, and emission scenarios: According to Wang and Xiong [30], the ECHAM4 and the HadCM2 prediction models produce better simulation results for ground temperature and rainfall in East Asia than the other prediction models. However, the TYN SC2.0 data set did not include the HadCM2 model; therefore, the ECHAM4 prediction model was selected. In general, the standard period for climate data analysis is 30 years [31]; therefore, average climate data for the 2020s (2011–2040) were used for the simulation.

This model contains four greenhouse gas emission scenarios (A1FI, A2, B1, and B2), which differed in term of predictions of socioeconomic growth and new technologies [17]. Xiong [32] concluded that the B2 scenario (regional sustainable development) was closest to the likely future development in China. However, to compare the predicted results under the four scenarios, the present study used all four scenarios for analyses.

The IPCC Fourth Assessment Report presented four emission scenarios: A1, A2, B1, and B2. The A1FI and A2 scenarios assume that future societies will emphasize economic development, while B1 and B2 scenarios assume that society will emphasize environmental protection. In addition, the A1FI and B1 scenarios emphasize global economic development, whereas the A2 and B2 scenarios emphasize regional development [10]. Using data from the IPCC Special Report on Emission Scenarios, Xiong [25] analyzed the specific future development framework for China in combination with the Chinese national conditions. When the four scenarios were compared and analyzed, the authors concluded that the B2 scenario (regional sustainable development) most closely reflected China’s future development. The B1 and A1FI scenarios described other possible development pathways. The B1 scenario considered global sustainable development, whereas the A1FI scenario examined the rapid growth of the global economy. The A2 scenario considered rapid population growth, which varied widely according to projected increases in the Chinese population. Therefore, we primarily analyzed changes in potential distribution under the B2 scenario, and then compared the predictions made by the other scenarios with those made by the B2 scenario to obtain a comprehensive set of predicted results under all four scenarios.

Data conversion: The format of the TYN SC2.0 climate data was not compatible with the data set in CLIMEX; thus, the future climate data format was converted before data were entered into CLIMEX. Data were divided into three categories: temperature, relative humidity, and rainfall. The first two categories were calculated using original climate data, whereas the format of the rainfall data had to be converted. The “temperature” and “relative humidity” data were converted using methods described by Stephens et al. [31] and Mika et al. [33].

#### Known distribution of *R*. *ferrugineus*

**Fig 1:**
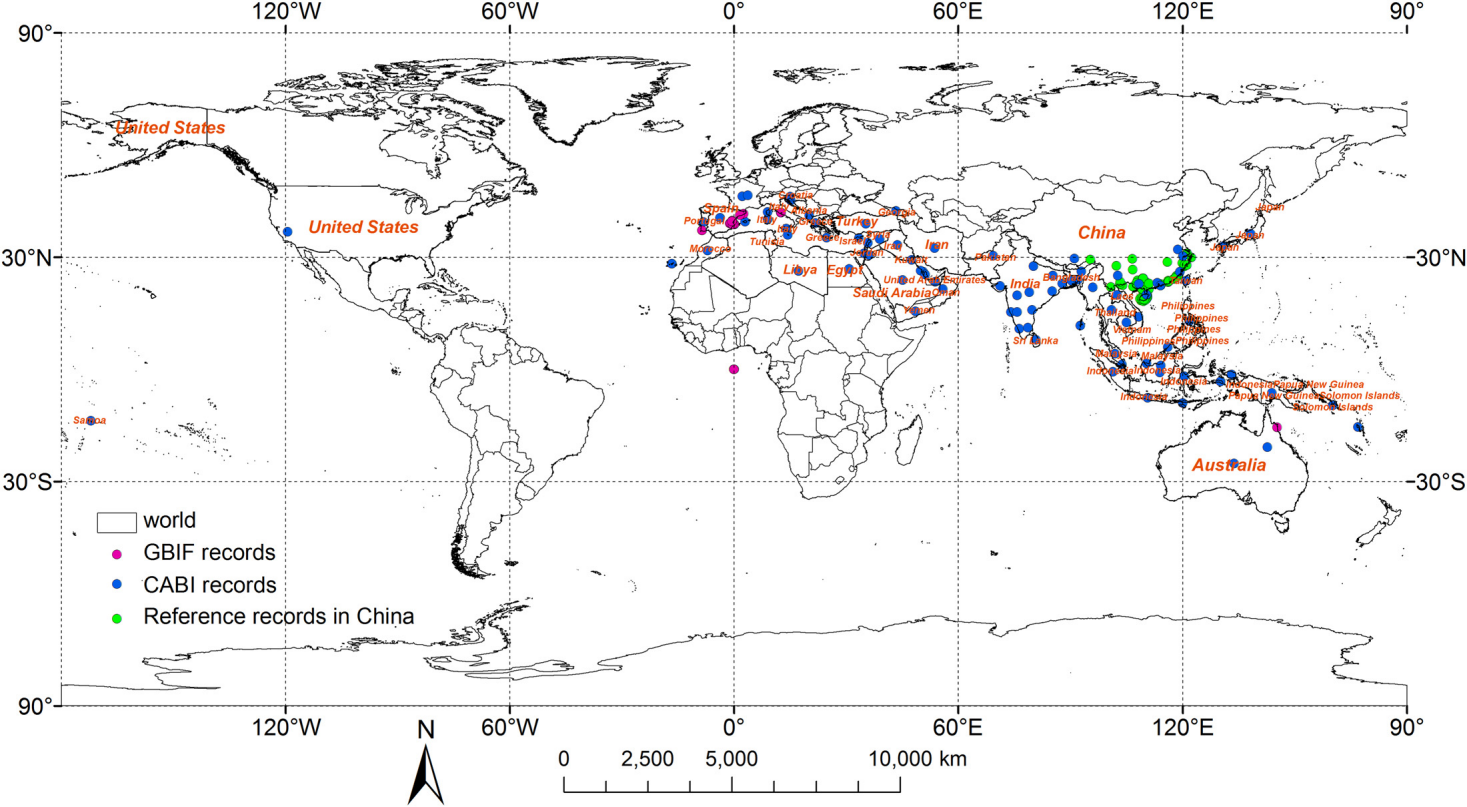
World known distribution of *Rhynchophorus ferrugineus*.

The known distribution of *R*. *ferrugineus* was recorded in CABI (S1 File) (http://www.cabi.org/cpc) and GBIF (http://www.gbif.org/species); these data were supplemented by data from the literature regarding the known distribution in China (S2 File). The worldwide distribution of *R*. *ferrugineus* is shown in Fig 1. *R*. *ferrugineus* was present in 59 countries, including those in Europe, Asia, and Africa.

#### Biological information

Temperature: Ou et al. [34] studied the effects of different constant temperatures on the growth and development of *R*. *ferrugineus* in Guangxi Province, and linear regressions determined the developmental threshold temperatures (± SE) for pre-oviposition, the egg, larva, pupa, and the entire generation. The temperatures were 16.11 ± 1.44, 14.10 ± 0.56, 15.28 ± 0.21, 14.89 ± 0.24, and 14.15 ± 0.54°C, respectively. The effective accumulated temperatures (± SE) for the above stages were 12.49 ± 2.04, 31.86 ± 1.65, 748.60 ± 15.79, 417.94 ± 20.80, and 1215.50 ± 28.56 degree days, respectively. Li [35] reared *R*. *ferrugineus* under seven constant temperatures (16, 20, 24, 28, 32, 36 and 40°C). The developmental threshold temperature (± SE) and effective accumulated temperature (± SE) for the entire generation were 17.41 ± 1.45°C and 1590.72 ± 193.78 degree days, respectively. The eggs failed to survive at 16°C and 40°C during the experiment. To clarify the effect of temperature on the development of *R*. *ferrugineus*, Zhao and Ju [36] used five constant temperatures (19, 22, 26, 30 and 33°C) in the laboratory, and the developmental threshold temperature and the effective accumulated temperature required for the entire generation were 14.2°C and 1067.7 degree days, respectively. They also reported that the most suitable temperature range for the development of *R*. *ferrugineus* was from 26°C to 30°C. Salama and Hamdy reported that the developmental threshold temperatures for egg and larva were -1.78°C and 18.6°C, respectively, and they observed hibernating larvae when the temperature was below 18.6°C [35]. Salama et al. [37] found that the minimum and maximum developmental threshold temperatures were -2.3°C and 44–45°C, respectively. Ju et al. [26] determined the supercooling points (SCPs) with the supercooling point determinator and the cold tolerance in environmental chambers at low temperatures, and they reported that mean supercooling points (± SE) of the egg, 1^st^ instar larva, 5^th^ instar larva, 9^th^ instar larva and adult were -5.92 ± 0.97, -6.42 ± 1.38, -7.19 ± 1.59, -7.43 ± 1.65 and -11.84 ± 1.45°C, respectively. For 72 h at low temperature, the Ltemp_5_0 (semilethal temperature) for the above stages was 1.61, -1.67, -2.39, -2.40 and -0.40°C, respectively. She and Feng [38] measured the mean supercooling points (± SE) of the adult, larva and pupa of the pest in Lishui and found values of -7.44 ± 2.971, -3.35 ± 0.596 and -2.74 ± 1.483°C, respectively.

Humidity: Huang et al. [39] concluded that the climatic conditions had an obvious influence on the trapping effect of pheromones for *R*. *ferrugineus*, and the trapped population was significantly reduced in rain and low temperatures. This suggested that *R*. *ferrugineus* preferred to live in a low temperature and a low humidity environment. Based on two irrigation methods (drip irrigation and flood irrigation) in the forest, Aldryhim and Al-Bukiri studied the distribution of *R*. *ferrugineus* and found that more hosts were harmed in the areas with flood irrigation than in the areas with drip irrigation, which led them to conclude that soil moisture was an important factor that influenced the infection and spread of *R*. *ferrugineus*. Aldryhim and Khalil examined the survival of *R*. *ferrugineus* in a dry peat bog, a wet peat bog and water. They found that in the dry peat bog the adults lived up to 2.5 d, whereas they lived up to 39.5 d and 23.5 d in the wet peat bog and water, respectively [35].

### Research Methods

#### Overall analytical process

**Fig 2:**
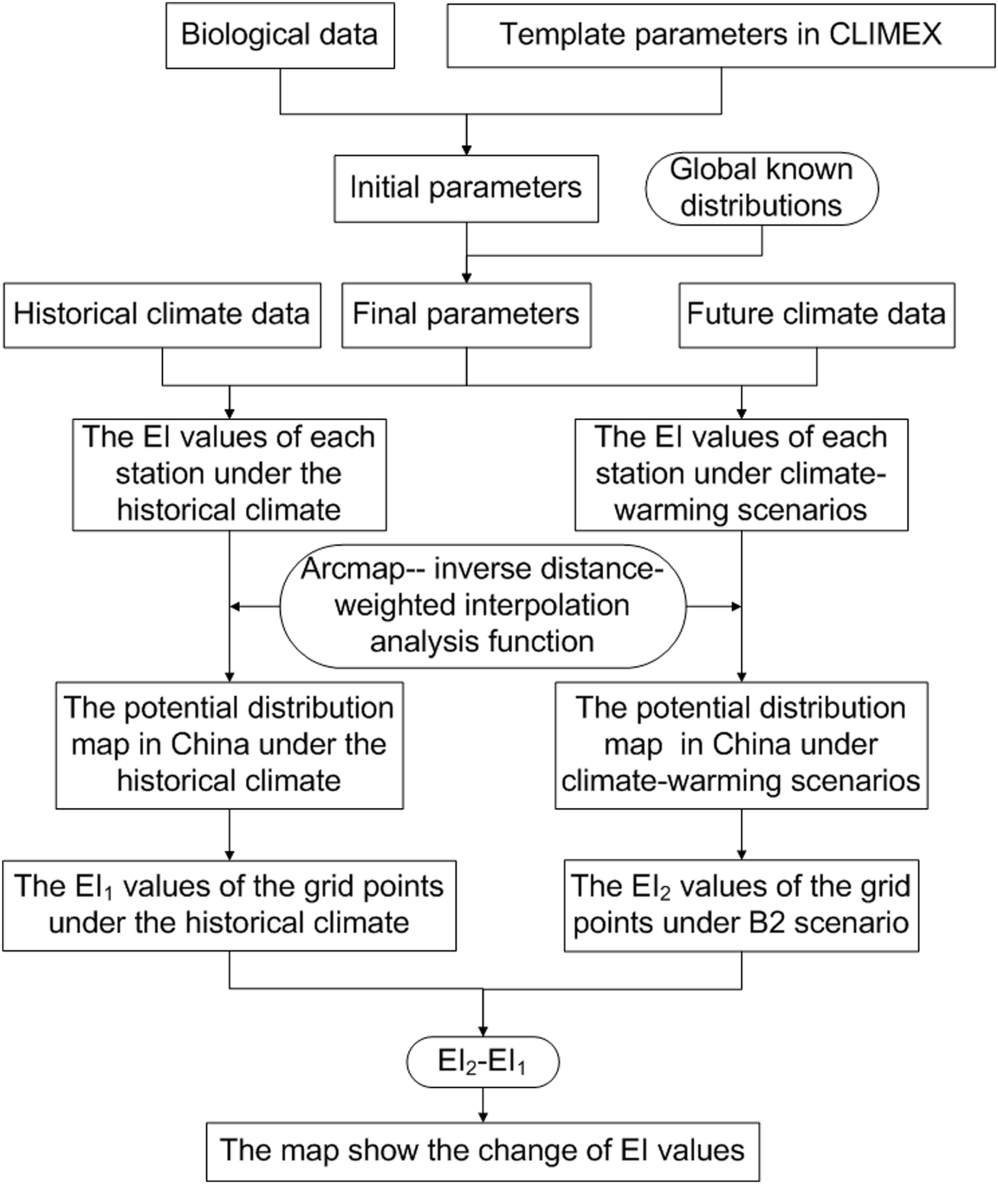
Flowchart indicating the process of the study.

An overview of the analytical process is presented in Fig 2.

#### Selection of CLIMEX parameters for *R*. *ferrugineus*

**Fig 3:**
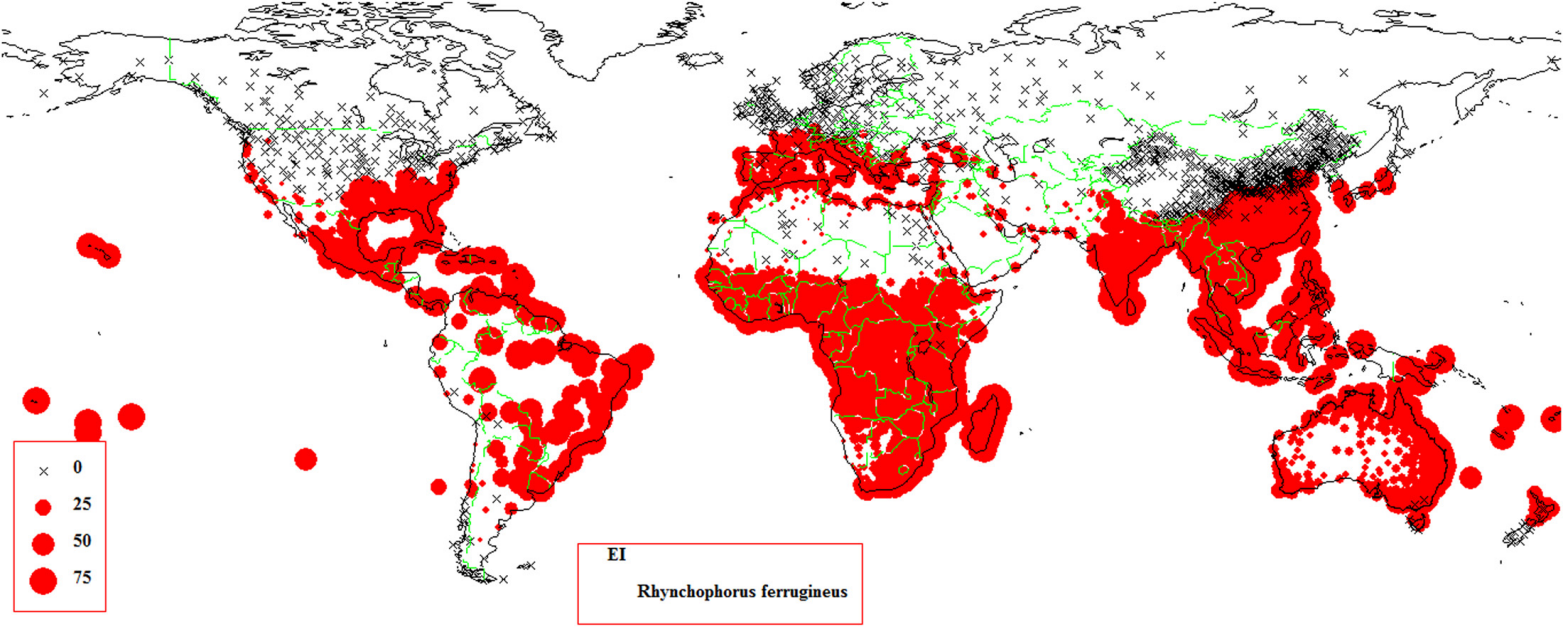
Predicting results for *Rhynchophorus ferrugineus* created by CLIMEX.

**Fig 4:**
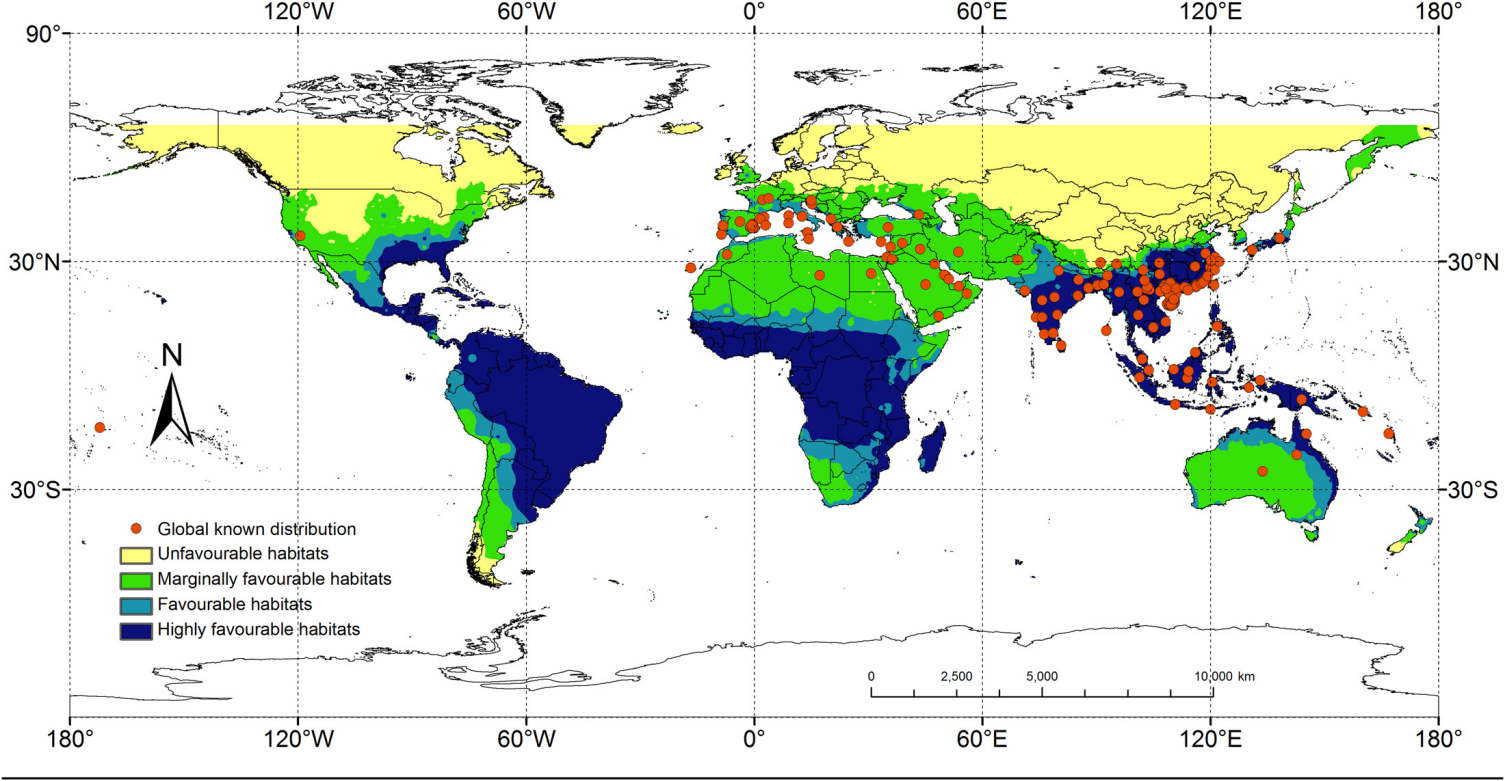
Global predicted potential distribution of *Rhynchophorus ferrugineus* under current climate.

The three templates (wet tropical, temperate, and desert) in CLIMEX (which are in accordance with the distribution zones of *R*. *ferrugineus*), were chosen as references when *R*. *ferrugineus* was created as a new species in CLIMEX. The initial parameters were used to predict potential distribution and to compare predicted distribution with known distribution: the parameters were then debugged based on these comparisons. The parameters required for *R*. *ferrugineus* survival (Table 1) were defined when the result for the world climate predicted by CLIMEX (Fig 3) was consistent with the actual distribution (Fig 4). The debugging process is described below.

**Table 1:** CLIMEX parameter values for *Rhynchophorus ferrugineus*.

CLIMEX parameter	Wet tropical template	Desert template	Temperate template	Final parameter
DVO-Lower temperature threshold	15	15	8	10.3
DV1-Lower optimum temperature	26	25	18	28
DV2-Upper optimum temperature	33	40	24	32
DV3-Upper temperature threshold	36	44	28	40
PDD-Effective accumulated temperature	0	0	600	1100
TTCS-Cold stress temperature threshold	0	2	0	–2.3
THCS-Cold stress temperature rate	0	0.001	0	0.05
TTHS-Heat stress temperature threshold	36	44	30	44
THHS-Heat stress temperature rate	0.001	0.001	0.005	0.001
SMO-Lower soil moisture threshold	0.3	0	0.25	0
SM1-Lower optimal soil moisture	0.5	0.001	0.8	0.5
SM2-Upper optimal soil moisture	1.75	0.2	1.5	1.75
SM3-Upper soil moisture threshold	2	0.3	2.5	2
SMDS-Dry stress threshold	0.3	0	0.2	0
HDS-Dry stress rate	0.006	0	0.005	0.006
SMWS-Wet stress threshold	2	0.3	2.5	2
HWS-Wet stress rate	0.002	0.1	0.002	0.002

When setting the parameters, the SI was first debugged to define the borders of the potential distribution and to define the specific distribution based on the GI and limiting conditions. The minimum and maximum developmental threshold temperatures found by Salama et al. [37] were used as the TTCS and HS temperature thresholds (TTHS), which were -2.3°C and 44°C, respectively. Based on the northern boundary of the known distribution of the weevil in China, the CS temperature rate (THCS) was set at 0.05, whereas the HS temperature rate (THHS) was set at 0.005. *R*. *ferrugineus* prefers to live in wet environments [35, 39]; therefore, the wet tropical template (which was aimed at hygrophilous species) was used as the primary reference when setting the dry (wet) SI. However, *R*. *ferrugineus* is highly adaptable and survives in dry environments with a measure of drought tolerance, as reported in Libya and Egypt (http://www.cabi.org/). Therefore, the DS threshold (SMDS) was defined as zero after being debugged repeatedly, and the other parameters of dry (wet) stress were in set accordance with the wet tropical template data.

The temperature indices were debugged according to the actual distribution using biological data as references. Based on the experimental results of Li [35] and Salama et al. [37], the optimum temperature range (DV1–DV2) was defined as 28–32°C, with the upper temperature threshold (DV3) at 44°C. The experimental results of Ou et al. [34], Li [35], and Zhao & Ju [36] were used as the primary references for setting the lower temperature threshold (DV0) and the effective accumulated temperature (PDD). To include the northern boundary area in the potential distribution, the DV0 and PDD were set 10.3°C and 1100 degree days, respectively. When the other parameters of the GI were set, all the parameters were in accordance with the wet tropical template parameters, except that the soil moisture threshold (SM0) was set lower to zero.

#### Classification of EI values

The EI values were classified according to the actual conditions in order to describe the most suitable climates for the species in more detail. The standard of classification is species-dependent; thus, the standard must be defined in accordance with the different ranges of the EI values reflecting different degrees of occurrence of *R*. *ferrugineus*. Information about the worldwide distribution and occurrence of *R*. *ferrugineus* is difficult to obtain; therefore, only the distribution records on CABI and the degree of occurrence in China were used to classify EI values.

Of all the known distributions, the lowest EI value (0.29) was obtained from the station located in Motuo, Tibet. To make sure that the areas surrounding this station were marginally favorable, the interpolation created by the IDW interpolation function in ArcGIS was considered, and the EI value that divided the unfavorable and marginally favorable habitats was set at 0.2. Based on the distribution records on CABI, the maximum and minimum EI values for the areas with low level occurrence were 12.3 and 7.2, respectively. To ensure that the interpolation of the potential distribution was in accord with the records in CABI, the cutoff value for the marginally favorable and favorable habitats was set at 13.0. When setting the cutoff value for the favorable and highly favorable habitats, the maximum EI value of the favorable habitats was debugged so that the degree of suitability of these locations was in accordance with the actual conditions. However, when the EI value of a location was more than 30, implying that it was very suitable for species survival [40], the cutoff value for favorable and highly favorable habitats was set at 30. Finally, the EI values were grouped into four arbitrary classes: unfavorable (0–0.19), marginally favorable (0.20–12.99), favorable (12.99–29.99) and highly favorable (≥ 30) habitats. The map, which showed the degree of suitability of both the world and known distributions (Fig 4), proved the validity of the EI value classifications.

#### Parameters test

According to the records in the reference, *R*. *ferrugineus* was primarily distributed throughout the provinces of Hainan, Guangdong, Shanghai, Fujian, Sichuan, Zhejiang, Taiwan, Yunnan, Hong Kong, Guangxi, Tibet, Guizhou, Chongqing, and Jiangsu [26, 41, 42]. Invasion of *R*. *ferrugineus* into the provinces of Hainan, Guangdong, Shanghai, Fujian, Yunnan, Hong Kong, Guangxi, Chongqing, and Jiangxi caused huge losses. As shown by comparison with the results in Table 2, the results predicted by the CLIMEX parameters were in accordance with the actual distribution conditions.

**Table 2:** EI^1^ value for each province in China (calculated by CLIMEX).

Province	No^2^. of stations in CLIMEX	No. of highly favorable stations	No. of favorable stations	No. of marginally favorable stations	No. of unsuitable stations	Average EI value for the station	Maximum EI value	Minimum EI value
Anhui	25	24	1	0	0	40.3	45.9	0
Beijing	3	0	0	0	3	0.0	0	0
Fujian	29	27	2	0	0	51.2	63.2	21.9
Gansu	32	0	1	0	31	0.7	20.8	0
Guangdong	36	36	0	0	0	62.9	74.9	50.6
Guangxi	25	25	0	0	0	59.1	74.6	43.9
Guizhou	34	29	5	0	0	38.7	54.8	17.8
Hainan	9	9	0	0	0	75.7	86.3	66.5
Hebei	21	0	0	0	21	0.0	0	0
Henan	20	7	3	4	6	17.9	42.5	0
Heilongjiang	36	0	0	0	36	0.0	0	0
Hubei	35	33	1	0	1	42.0	46.7	0
Hunan	34	33	1	0	0	45.2	51	24.1
Jilin	32	0	0	0	32	0.0	0	0
Jiangsu	23	19	4	0	0	36.5	43.8	13.9
Jiangxi	28	27	1	0	0	46.6	54.9	25.8
Liaoning	27	0	0	0	27	0.0	0	0
Inner Mongolia	49	0	0	0	49	0.0	0	0
Ningxia	12	0	0	0	12	0.0	0	0
Qinghai	39	0	0	0	39	0.0	0	0
Shandong	33	0	7	1	25	5.4	28.7	0
Shanxi	28	0	0	0	28	0.0	0	0
Shaanxi	37	8	4	1	24	9.8	39.4	0
Shanghai	2	2	0	0	0	44.3	44.9	43.7
Sichuan	50	26	7	1	16	24.5	48.8	0
Tianjin	4	0	1	0	3	5.6	22.3	0
Tibet	38	0	1	0	37	0.7	24.9	0
Xingjiang	68	0	0	0	68	0.0	0	0
Yunnan	34	25	7	0	2	36.7	64.5	0
Zhejiang	27	27	0	0	0	45.4	52.3	41.4
Chongqing	15	14	0	0	1	42.4	49.3	0
Taiwan	2	2	0	0	0	68.0	71	65
Hong Kong	1	1	0	0	0	50.0	50	50
Total	888	374	46	7	461	25.7	86.3	0

^1^EI = Ecoclimatic Index of CLIMEX.

^2^No = Number

## Results

### Potential Distribution for *R*. *ferrugineus* According to Current Climate Data

**Fig 5:**
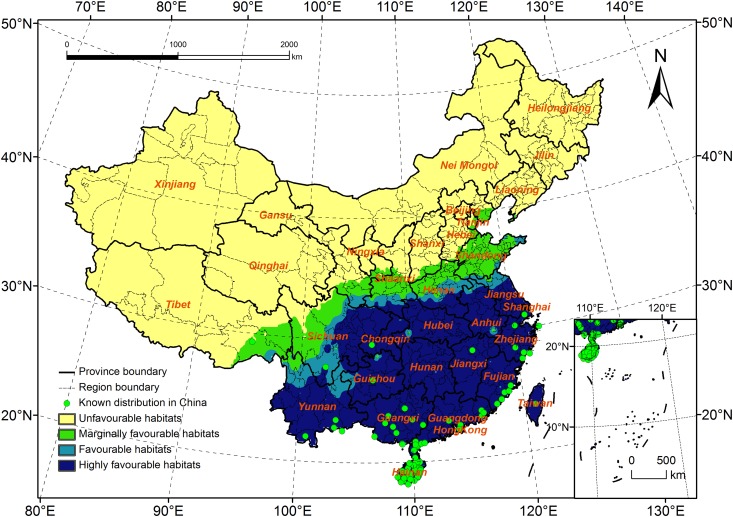
Potential distribution for *Rhynchophorus ferrugineus* under the current climate (1981–2010).

Based on current climate data for 1981–2010, the EI values of each station in China were calculated and imported into ArcGIS. Finally, the potential distribution of *R*. *ferrugineus* under these conditions was created using the IDW interpolation function (Fig 5). In China, the total potential distribution of *R*. *ferrugineus* was approximately 3.01 million km^2^, which represents 31.3% of the total land area. The estimated distribution range covered 18.2–40.1°N and 93.7–122.7°E, and was primarily located in southern China.

Highly favorable habitats: The area of these habitats amounted to 2.07 million km^2^, which is 21.6% of the total land area and 68.9% of the total potential distribution. All the southern areas in China were highly favorable habitats, including the provinces of Hainan, Guangxi, Guangdong, Hong Kong, Macao, Hunan, Jiangxi, Fujian, Yunnan, Guizhou, Hubei, Anhui, and Jiangsu, and most areas of Taiwan, southeast Sichuan, and southern areas of Shaanxi and Henan.

Favorable habitats: The area of these habitats amounted to 0.32 million km^2^, which is 3.3% of the total land area and 11.0% of the total potential distribution. These habitats spread to the north along the boundary of the highly favorable habitats. From west to east and south to north, the local areas of Southeast Tibet, North and Northeast Yunnan, South and Northeast Sichuan, South Shaanxi, central Henan, north of Anhui and Jiangsu, East Shandong, and local areas of Taiwan were all locations with favorable habitats.

Marginally favorable habitats: The area of these habitats amounted to 0.62 million km^2^, which is 6.4% of the total land area and 20.1% of the total potential distribution. These habitats were primarily located in Southeast Nyingchi and South Changdu in Tibet, South Garze, most areas of Ngawa in Sichuan, South Gansu, Central Shaanxi, Northwest Henan, Southeast Shanxi, most areas of Shandong and Tianjing, and the local areas of Hebei.

In conclusion, the potential distribution of *R*. *ferrugineus* in China according to current climate data included the provinces of Hainan, Yunnan, Guangdong, Guangxi, Hong Kong, Guizhou, Hunan, Jiangxi, Fujian, Taiwan, Sichuan, Chongqing, Hubei, Anhui, Shanghai, Zhejiang, Jiangsu, Tibet, Gansu, Shaanxi, Shanxi, Henan, Shandong, and Hebei. *R*. *ferrugineus* is not yet present in Hunan, Hubei, Anhui, Jiangsu, Gansu, Shaanxi, Shanxi, Henan, Shandong, and Hebei; however, some are highly favorable habitats for *R*. *ferrugineus* (e.g., Anhui and Hunan). When the pest invades these areas, huge outbreaks might occur.

### Potential Distribution of *R*. *ferrugineus* According to Future Climate Warming Scenarios

We next predicted the future potential distribution of *R*. *ferrugineus* in China under four emission scenarios (A1FI, A2, B1, and B2). The details are summarized in Table 3. Compared with the distribution according to current climate data, the areas with highly favorable habitats increased under all four scenarios. The number of ranges (moving from small to large) will increase under the A2, B2, B1, and A1FI scenarios, and in that order. The areas of favorable and marginally favorable habitats will decrease, and the total area of the potential distribution will decrease to a certain extent, except for that predicted by the A1FI scenario.

**Table 3:** Area changes in the potential distribution of *Rhynchophorus ferrugineus* predicted under the four scenarios in the ECHAM4 model.

Model case	Highly favorable habitats		Favorable habitats		Marginally favorable habitats		Unfavorable habitats		Total potential distribution	
	Area/mil^1^. km^2^	Percentage	Area/mil. km^2^	Percentage	Area/mil. km^2^	Percentage	Area/mil. km^2^	Percentage	Area/mil. km^2^	Percentage
Historical climate	2.07	21.6%	0.32	3.3%	0.62	6.4%	6.61	68.7%	3.01	31.3%
A2 scenario	2.21	23.0%	0.24	2.5%	0.39	4.1%	6.76	70.4%	2.85	29.6%
B2 scenario	2.25	23.4%	0.25	2.6%	0.40	4.2%	6.71	69.8%	2.90	30.2%
B1 scenario	2.35	24.4%	0.26	2.7%	0.38	4.0%	6.62	68.8%	2.99	31.2%
A1FI scenario	2.39	24.8%	0.27	2.8%	0.39	4.0%	6.57	68.4%	3.04	31.6%

^1^mil = million.

### Comparison of the Potential Distribution of *R*. *ferrugineus* under the B2 Scenario with that under Current Climate Data

**Fig 6:**
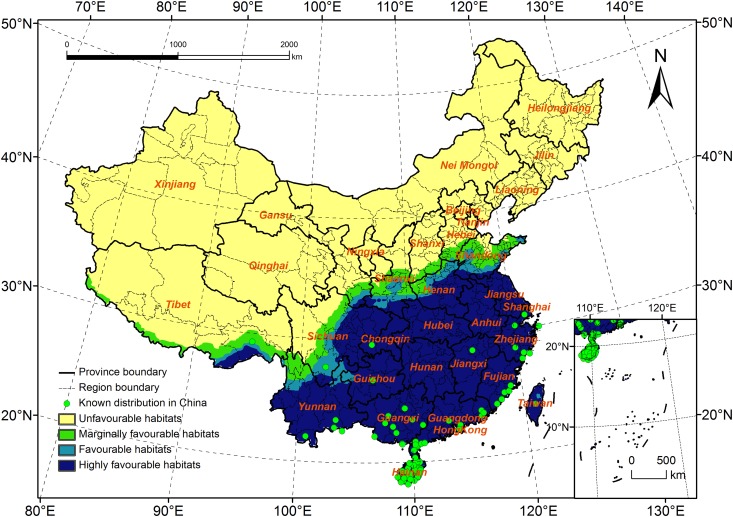
Potential distribution for *Rhynchophorus ferrugineus* under B2 scenario (2020s).

As it most likely reflects China’s future development, the potential distribution under the B2 scenario might be more accurate than that predicted by the other scenarios. Under this scenario, the total area of potential distribution will decrease by 0.11 million km^2^. The area of highly favorable habitats will increase by 0.18 million km^2^, whereas the areas of favorable and marginally favorable habitats will decrease by 0.07 and 0.22 million km^2^, respectively. The estimated distribution range will cover 18.2–37.9°N and 78.8–122.7°E (Fig 6).

**Fig 7:**
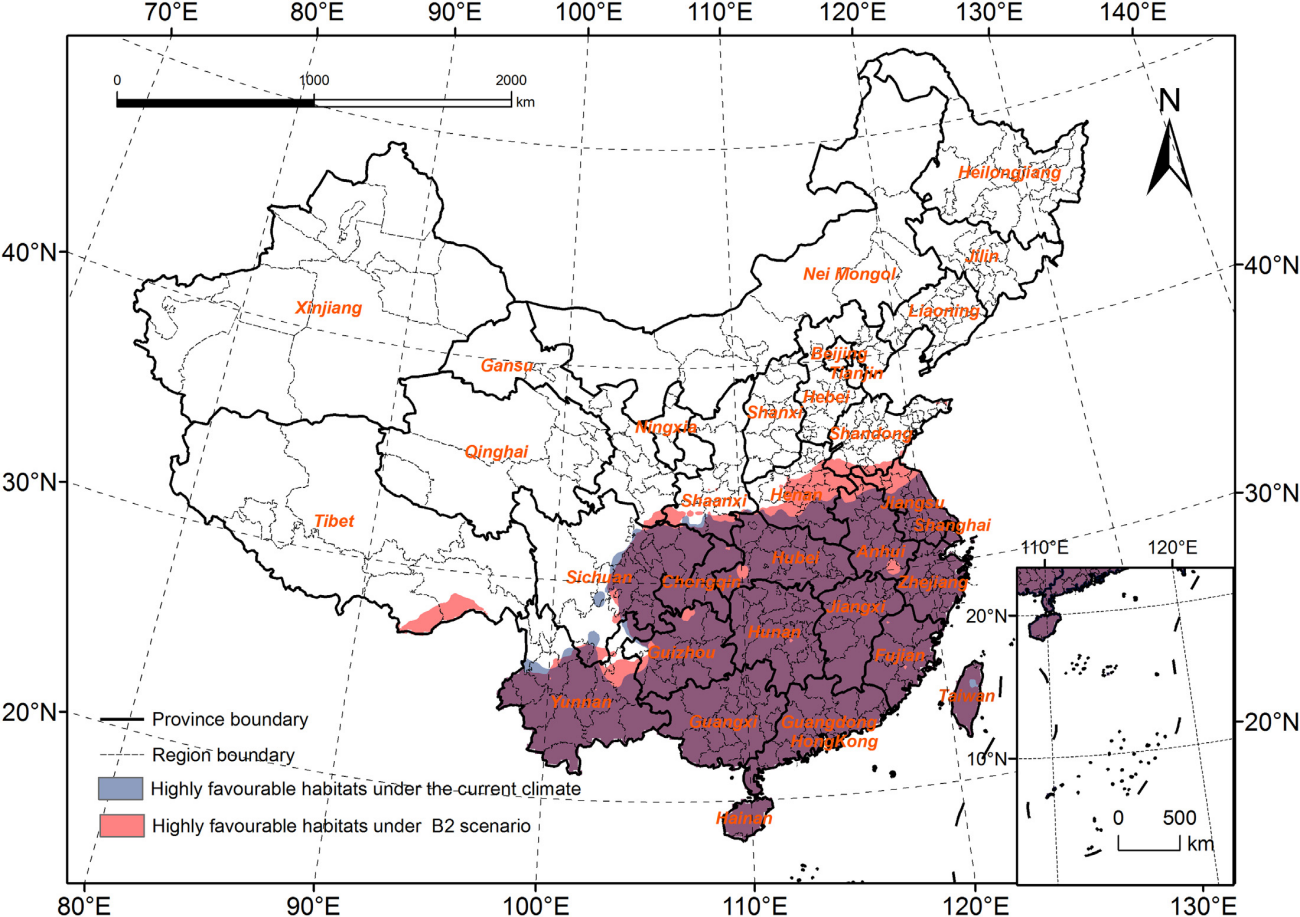
Comparison of the highly favourable habitats under the current climate and B2 scenario.

The boundary of the highly favorable habitats will expand to the north in Tibet, and in the Henan and Shandong Provinces, and the northern boundary will spread to South Shandong (Fig 7). In Tibet, the North of Nyingchi and Shannan will change from marginally favorable habitats to highly favorable habitats. In Yunnan, the highly favorable habitats will spread to the North of Qujing, and in Sichuan, the habitats will expand to the junction of three provinces (Sichuan, Gansu and Shanxi). In Henan, the habitats will spread to the north, and most of the area in this province will be highly suitable for *R*. *ferrugineus* survival.

**Fig 8:**
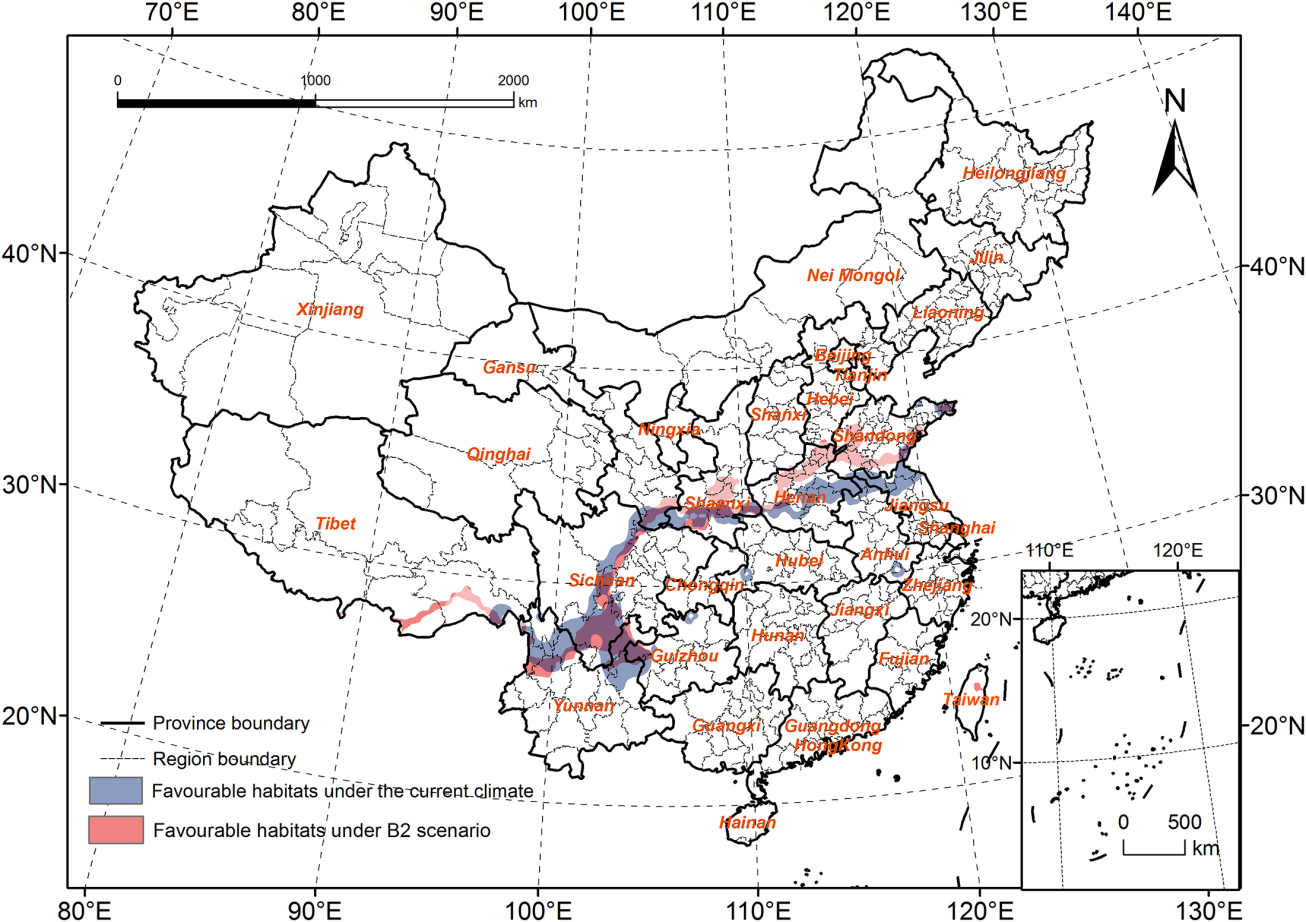
Comparison of the favourable habitats under the current climate and B2 scenario.

The area of favorable habitats will clearly decrease (Fig 8); the habitats also had a tendency to spread to the north in the provinces of Tibet, Shaanxi, Henan, and Shandong. However, in Sichuan and Yunnan Provinces, it will spread to the South, and most areas of Dali and Lijiang will change from highly favorable habitats to favorable habitats.

**Fig 9:**
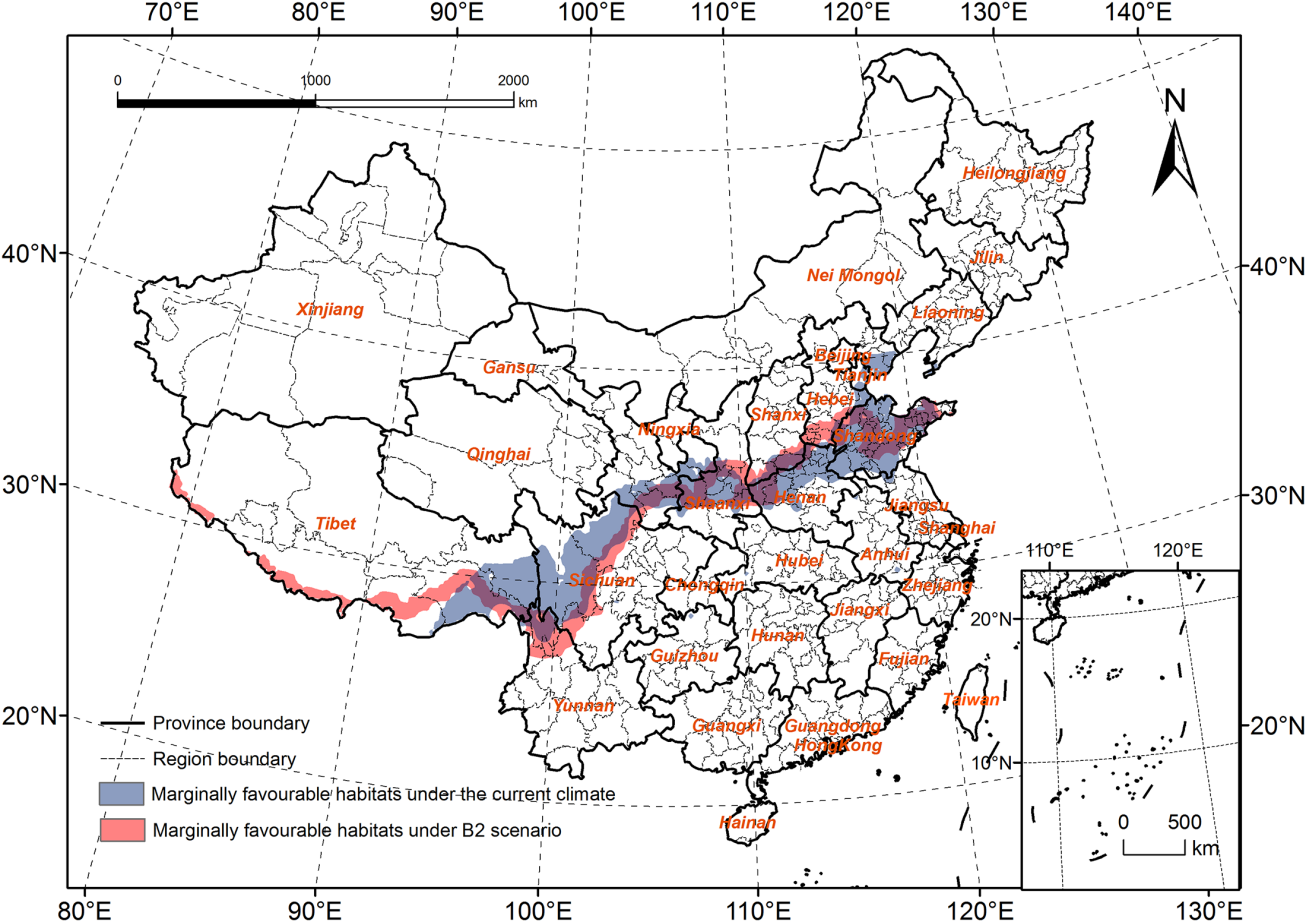
Comparison of the marginally favourable habitats under the current climate and B2 scenario.

The marginally favorable habitats had a tendency to spread to the south in China, and the area decreased significantly (Fig 9). In Tibet, the edges of Southwest Ngari and Shigatse will change from unfavorable habitats to marginally favorable habitats. The area of marginally favorable habitats will greatly decrease in Nyingchi, Shannan, and Changdu; in Sichuan, these habitats will decrease in the south. In Gansu, the northern boundary of the habitats will move south, whereas the southern boundary will not change much. In Shanxi and Hebei, both the southern and northern boundaries will expand to the north, but the expanded range of the southern boundary will be larger than that of the northern boundary, which will cause the overall area to decrease. The marginally favorable habitats in Hebei, East Tianjin, and North Shandong will become unfavorable habitats.

### Comparison of the Potential Distribution of *R*. *ferrugineus* under the B2 Scenario and Other Scenarios

**Fig 10:**
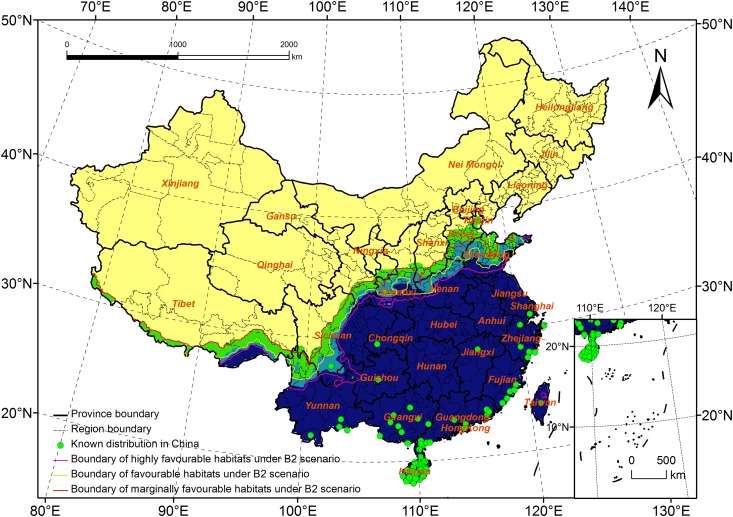
Potential distribution for *Rhynchophorus ferrugineus* under A1FI scenario (2020s).

A1FI and B2 scenarios: Compared with that under the B2 scenario, the potential distribution under the A1FI scenario will expand to the north, and the estimated distribution range will cover 18.2–39.6°N and 78.8–122.7°E, with the northernmost latitude of the potential distribution being 1.7° greater than that in the B2 scenario (Fig 10). As for the area of potential distribution, the A1FI scenario was the only one of the four that predicted an increase in area relative to that predicted using current climate data. The potential distribution area measured over 3 million km^2^, which was 0.03 million km^2^ more than that calculated from current climate data and 0.14 million km^2^ more than predicted by the B2 scenario.

Among the four scenarios, the A1FI scenario predicted the largest area of highly favorable habitats, 2.39 million km^2^, which was 0.32 million km^2^ more than that calculated from the current climate data and 0.14 million km^2^ more than predicted by the B2 scenario. The primary change was an expansion of the northern boundary to the north in Shaanxi and Shandong Provinces. In Shaanxi, it will expand to the south of Weinan and Xi’an. In Shandong, it will expand to Qindao and to the southwest of Weifang. However, the highly favorable habitats will not change much in the other provinces.

The area of favorable habitats was 0.02 million km^2^ more than the B2 scenario, and the northern boundary had a tendency to expand north in the south of Shaanxi, the west of Henan and the south of Shandong.

When the A1FI scenario was compared with the B2 scenario, we found that the area of marginally favorable habitats fell by 0.01 million km^2^. From west to east and from Tibet to Gansu, the change in habitats was not obvious. However, they are predicted to expand north and spread to Linfeng in Shanxi, to Langfang in Hebei, and to the other northern locations (Dongying and Bingzhou). Central Tianjing will change from an unfavorable habitat to a marginally favorable habitat.

**Fig 11:**
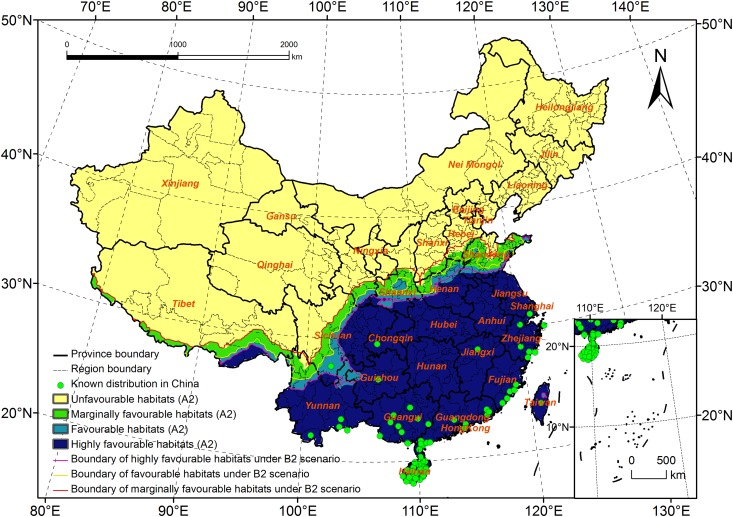
Potential distribution for *Rhynchophorus ferrugineus* under A2 scenario (2020s).

A2 and B2 scenarios: The potential distribution predicted by the B2 scenario was similar to that predicted by the A2 scenario. The overall change was not clear. The estimated distribution range was in accordance with the B2 scenario, i.e., 18.2–37.9°N and 78.8–122.7°E (Fig 11). The area of potential distribution predicted under the A2 scenario was 2.85 million km^2^, 0.05 million km^2^ less than that predicted by the B2 scenario. The area of highly favorable habitats will decrease by 0.04 million km^2^, and the area of both favorable and marginally favorable habitats will decrease by 0.01 million km^2^. In Henan and Shandong, the northern boundary of the highly favorable and favorable habitats will expand to the south. The northern boundary of marginally favorable habitats will expand to the north in Shanxi, Hebei and Shandong Provinces. The changes in the other provinces were in accordance with the B2 scenario.

**Fig 12:**
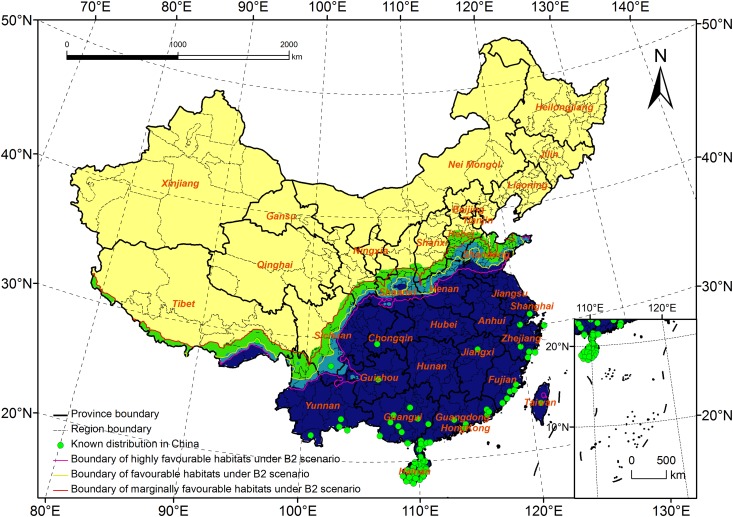
Potential distribution for *Rhynchophorus ferrugineus* under B1 scenario (2020s).

B1and B2 scenarios: Compared with that predicted by the B2 scenario, the entire potential distribution predicted under the B1 scenario will expand to the north, and the estimated distribution range is 18.2–38.7°N and 78.8–122.7°E, with the northernmost latitude of the potential distribution being 0.8° more than that in the B2 scenario (Fig 12). The potential distribution area under the B1 scenario was nearly 3 million km^2^, 0.02 million km^2^ less than that under the B2 scenario. The area of highly favorable and favorable habitats will increase by 0.10 million km^2^ and 0.01 million km^2^, respectively. However, the area of marginally favorable habitats will decrease by 0.02 million km^2^. The predicted potential distribution under the B1 scenario was similar to that under the A1FI scenario, and the change was the same.

In conclusion, the four emission scenarios showed little difference in their predictions compared with those based on current climate data. The primary predictions were that highly favorable habitats would increase and expand to the north in China, whereas both favorable and marginally favorable habitats would decrease.

Among the four scenarios, the B2 scenario most represented Chinese national conditions, whereas the A1FI scenario was the most harmful for the environment and showed the largest predicted distribution range. However, each of the four scenarios had value in terms of reference. If it is assumed that Chinese national conditions will not change significantly, then the B2 scenario is most consistent with the developmental trend of a future China. Thus, the predictions under the B2 scenario would be the most suitable for the control and quarantine of *R*. *ferrugineus*. Additionally, only small differences were found among the four predictions made under the four scenarios. In practice, the predictions made under the B2 scenario should be primary, whereas the predictions made under other scenarios should be complementary; thus, the predictions should be more accurate and preventive measures more effective.

### Analysis of the Variation in EI Values

**Fig 13:**
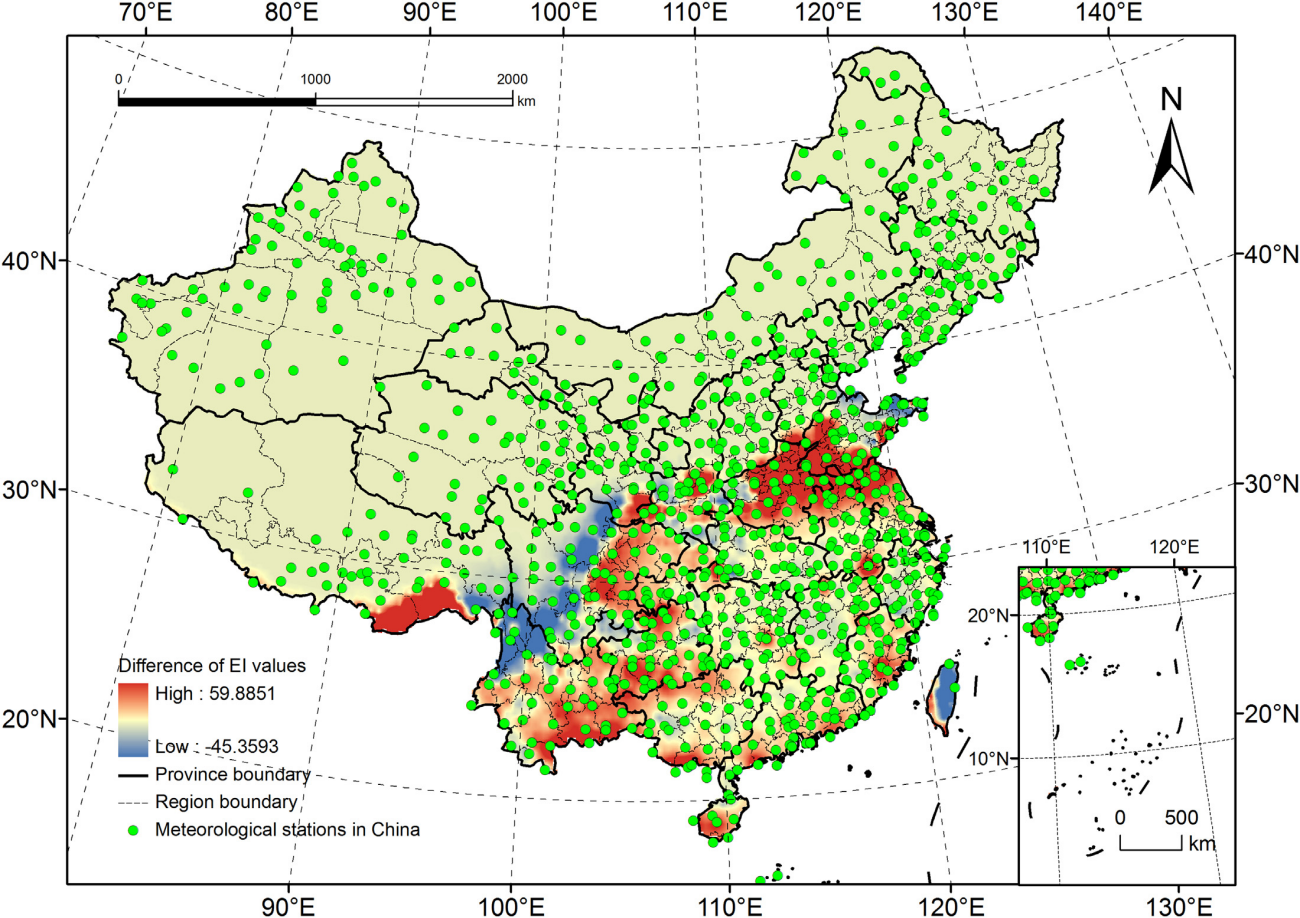
Difference values of the EI values under B2 scenario and current climate.

The difference (EI_2_-EI_1_) between the grid point EI values under the B2 scenario (EI_2_) and the current climate (EI_1_) was calculated using ArcGIS, and a map of EI value variation was created (Fig 13). The EI values did not increase in all areas, and the difference in EI values in some areas was very large, reaching 40–60. Twenty points were randomly selected in areas in which the variation in EI values was significant (the selected points had values less than 20 in some areas, which was a relatively small area). Then, the changes in the CLIMEX parameters (GI, MI and CS) in the different areas were compared to identify the reasons for the variation in EI values.

In most areas (shown in red in Fig 13), the EI value is predicted to increase greatly (as high as 59.9). We identified two factors that may be responsible. One is the density of the site distributions. Few meteorological stations were present in some areas, the accuracy of the EI values after interpolation would be influenced by the meteorological stations in remote areas. For example, no meteorological stations were present in the red areas of Tibet; thus, the EI values in these areas could only be interpolated based on the EI values in the surrounding areas. However, these areas were not suitable for *R*. *ferrugineus* survival; therefore, the EI values after interpolation based on current climate data, would be low than the practical values. Whereas the future climate data comprised grid data with a high resolution (0.5° × 0.5°), there were more detail future climate data in red areas climate data, and the EI values after interpolation would be more accurate and have big difference with the results under current climate. The other factor is climate change. As shown in Table 4, the GI and MI values (which are influenced by increases in temperature and humidity associated with global warming) will increase in provinces such as Guangdong, Guangxi, and Yunnan, resulting in higher EI values. In areas located around the borders of the potential distribution areas (i.e., the provinces of Anhui, Gansu, and Shandong), the main factor limiting the EI value was the CS; with temperatures rising, the cold stress that *R*. *ferrugineus* suffers in these locations will decrease, causing the EI values to increase.

**Table 4:** Change in the primary indices of the provinces in which EI values will increase.

Province	No. of stations	Type of climate	Average EI value	Average GI value	Average MI value	Average CS value
Anhui	20	B2 scenario	39.5	39.5	87.4	0.1
		Historical climate	24.3	36.4	77.1	49.4
Fujian	14	B2 scenario	54	54	100	0
		Historical climate	37.6	37.6	98.8	0
Gansu	12	B2 scenario	26.6	31.2	88.6	78.8
		Historical climate	12.4	24.6	68	358.7
Guangdong	10	B2 scenario	69.3	69.3	97.3	0
		Historical climate	57.5	58.8	85.7	0
Guangxi	20	B2 scenario	61.8	61.8	96.7	0
		Historical climate	48.2	48.5	88.7	0
Guizhou	20	B2 scenario	47.3	47.3	98.4	0
		Historical climate	31.9	34	98.7	25.8
Henan	20	B2 scenario	31.7	33.1	74	9.3
		Historical climate	10.4	30.9	60	141.2
Hubei	7	B2 scenario	38.2	38.2	99.9	0
		Historical climate	20.9	27.8	99.2	342.3
Hunan	6	B2 scenario	45.6	45.6	100	0
		Historical climate	33.4	33.4	100	0
Jiangsu	20	B2 scenario	39.4	40	89.3	1.6
		Historical climate	18.3	35	80.1	97.2
Jiangxi	4	B2 scenario	45.1	45.1	100	0
		Historical climate	32.7	32.7	100	0.1
Shandong	20	B2 scenario	24	33.8	70.6	31.3
		Historical climate	3.3	29.6	63.4	427.5
Shaanxi	20	B2 scenario	25.5	31.8	85.2	51.5
		Historical climate	4.2	24.1	62.3	283.9
Sichuan	20	B2 scenario	49.2	49.2	99.6	1.6
		Historical climate	37.8	38	86.5	74.7
Tibet	20	B2 scenario	42.6	43.9	81	39.8
		Historical climate	0.4	10.7	59.1	822
Yunnan	20	B2 scenario	57.1	57.1	90.1	0
		Historical climate	42.1	42.1	81.4	0

^1^EI = Ecoclimatic Index of CLIMEX.

^2^GI = Annual Growth Index Growth Indices of CLIMEX.

^3^MI = Moisture Index of CLIMEX.

^4^CS = Cold Stress of CLIMEX.

However, in some areas (shown in blue in Fig 13), the EI values will decrease by a large margin (up to 45.4), although the two responsible factors (site distribution density and climate change) remain the same. Although the temperature changes in China correlate with global changes, in some areas (or for a certain time) the phenomenon of extreme temperatures might decrease (Table 5), causing the GI to decrease and the CS to increase; this will ultimately leading to lower EI values (this might occur in some areas in Sichuan and Yunnan). Only two meteorological stations were based in Taiwan, and the EI values for these two stations were high under the current climate data model. This caused the EI value for each grid point to be high after interpolation, which may be the reason for the large differences between the EI values calculated under the two types of climate model.

**Table 5:** Change in the primary indices of the provinces in which EI values will decrease.

Province	No. stations	Model case	Average EI^1^ value	Average GI^2^ value	Average MI^3^ value	Average CS^4^ value
Shandong	20	B2 scenario	1.5	32.1	77.7	372
		Historical climate	12	27.5	61.2	262.1
Shaanxi	20	B2 scenario	15.7	28.3	91	169.1
		Historical climate	21.8	29.9	76.3	189.2
Sichuan	20	B2 scenario	6.5	14.5	73.3	608.4
		Historical climate	23.6	26.2	68	237.6
Taiwan	20	B2 scenario	44.2	46.8	99.2	0
		Historical climate	63.4	64.8	99.5	0
Tibet	15	B2 scenario	2.7	8.4	79.8	891.8
		Historical climate	13.9	17.3	67.3	417.3
Yunnan	20	B2 scenario	3.8	12.6	77	570.1
		Historical climate	19.2	21.1	65.8	227.1

^1^EI = Ecoclimatic Index of CLIMEX.

^2^GI = Annual Growth Index Growth Indices of CLIMEX.

^3^MI = Moisture Index of CLIMEX.

^4^CS = Cold Stress of CLIMEX.

## Discussion

### Effects of Climate Change on the Distribution Predictions

According to the predictions, the area of the total potential distribution did not always increase under the climate warming scenarios; however, all of the areas with highly favorable habitats showed different levels of increase, and the order of the increase was A2 < B2 < B1 < A1FI. The IPCC [10] reported the projected degree of global average surface warming at the end of the 21st century, and under the four scenarios the surface temperature of the earth would increase by different degrees in the future: the order of the increase would be B1 < B2 < A2 < A1FI. When the two orders of increase were compared, the change in temperature was not in accordance with the change in the area of highly favorable habitats, but they were similar. The CLIMEX model considers many factors when using climate data for predictions, including temperature, humidity, and rainfall, so the predictions would also be influenced by humidity and rainfall data. Additionally, climatic factors which effect the growth and development of insects were not all considered by CLIMEX. However, temperature and humidity have the greatest effect on insects; therefore, the predictions made by CLIMEX have a certain of level of dependability.

In addition to climatic factors, many other environmental factors, such as soil (particularly soil organisms), food, natural enemies, and human activities, were not considered when predicting the potential distributions; this was because of limitations associated with CLIMEX. Therefore, these environmental factors should be considered in the light of local conditions during practical application.

### Effects of Different Research Methods on the Predicted Distribution

Some researchers in China used different methods to predict the potential distribution of *R*. *ferrugineus* under current climate models.

Ju et al. used the “Compare Locations” function of CLIMEX to predict the potential distribution of *R*. *ferrugineus* in China [25]. The predicted range of the potential distribution was similar to the results of the present study; however, there were some differences regarding the degree of climatic suitability of the potential distribution. For example, Ju et al. regarded some provinces (i.e., Yunnan, Guizhou, and Hunan) were regarded as favorable habitats, whereas they were viewed as highly favorable habitats in our study. One explanation for this might be that different climate data were used. Ju et al. input the data set from the 85 Chinese meteorological stations into CLIMEX (climate data from 1961 to 1990). Here, we used the China Surface Climate Monthly Standard Values data set (1981–2010) from China meteorological data sharing service system (http://cdc.cma.gov.cn/), which contained data from 885 meteorological stations. We also input data from Taiwan and Hong Kong into CLIMEX to yield 888 climate data stations. Additionally, the methods used for predictions were different. The ‘Match Climate’ function used by Ju et al. only considers climate data, whereas the ‘Compare Locations’ function used in the present study bases its predictions on both climate and biological data.

Ju et al. [26] examined the cold tolerance in environmental chambers at low temperatures and simulated field overwintering in Shanghai. They determined that the northern limit for overwintering of *R*. *ferrugineus* was approximately 35°N. However, according to the minimum temperature data and the CS indices of the species, the present study determined the northern limit for overwintering *R*. *ferrugineus* (which coincided with the northern boundary of marginally favorable habitats) to be 40.1°N. Differences in the research approach were the most likely reason for this difference. Ju et al. used the Ltemp_5_0 of the overwintering stage to measure the cold tolerance and primarily referred to the climatic divisions of China to define that the northern limit for overwintering was approximately 36.5°N. However, here, we relied on the cold SI (TTCS and THCS) and used CLIMEX to calculate the CS of each area. In the locations with a CS > 100, cold stress was the most important factor limiting the survival of the species; therefore, the EI values were used to define the northern limit for overwintering. Both methods were based on the cold tolerance of the species, but the standards to define cold tolerance were different; this may be another explanation for the different results. Additionally, Ju et al. considered that the host plants (palms) of *R*. *ferrugineus* were primarily distributed to the south of the Qinling Mountains, with few distributed to the north [43], and finally defined the northern limit for overwintering as 35°N. However, we did not consider host distribution in this study.

Li et al. [24] used GARP to predict the potential distribution of *R*. *ferrugineus* in China, and the northernmost point of the distribution range reached 32°N. The pest could survive in parts of south Jiangsu, central Anhui, northern Hubei and northern Sichuan. Compared with the results predicted by GARP, the range of the potential distribution predicted by CLIMEX was large. The GARP model used a genetic algorithm to analyze 14 environmental factors associated with the known distribution, including temperature, humidity, altitude, and rainfall, and extrapolated the optimum environmental conditions for the species to survive. The CLIMEX model used the growth and development data of the species to analyze the environmental factors that influenced the establishment of the population, taking into account climate data relevant to the target areas when calculating the potential distribution. The breakthrough point of the two models was also different. The former focused on environmental factors, whereas the latter focused on the biological characters of the species; thus, the results predicted by the two approaches complemented each other [44].

### Effect of the Host on the Control and Quarantine of *R*. *ferrugineus*

When *R*. *ferrugineus* invaded China, the weevil caused serious harm to palm plants. The host distribution in China extends from approximately 40°S to 40N°. Sixteen genera of Palmae (including *Arenga*, *Calamus*, *Livistona*, *Pinanga*, *Rhapis*, and *Trachycarpus*, among others) exist in southeast and southwest China. The hosts of *R*. *ferrugineus* are primarily distributed in Hainan, Yunnan, Guangxi, Guangdong, Fujian, and Taiwan. Some palms growing in Guizhou, Hunan, Sichuan, Zhejiang, Jiangxi, Tibet, Hong Kong, and Macao are not widely distributed [45]. The predictions made in the present study under the current climate and future climate warming scenarios show that the potential distributions of *R*. *ferrugineus* largely match those of Palmae. However, the provinces neighboring the areas in which Palmae grow, which were predicted to be in the potential distribution range of *R*. *ferrugineus* (e.g., Anhui, Shandong, and Henan) harbored few hosts. As a distinctive ornamental tree species, palms have high landscape value; thus more research has been conducted on the introduction and domestication of Palmae in China [46–48]. The environment suitable for the host plants will change as the world warms; therefore, and the potential distribution of Palmae might spread northward.

The potential host distribution is liable to change and is difficult to predict due to external influences and climate change. Therefore, the distribution of the hosts in the region should be thoroughly investigated before initiating measures to control and quarantine potential distribution areas of *R*. *ferrugineus*. In regions with no hosts, prevention and quarantine measures could be reduced to save both manpower and cost, whereas quarantine measures could be strengthened and preventive measures improved in regions that do contain host plants.

## Conclusions

Here, we used current and future climate data to predict the potential distribution of *R*. *ferrugineus* and compared the results. We also predicted possible future distributions under four different emission scenarios. The results may guide future prevention and quarantine measures. Quarantine officers can then focus on controlling pests in areas predicted to become more suited to insect survival. Conversely, they can reduce their efforts in areas likely to become less suitable in future. However, when using the results of EI values changing to guide the actual work, the areas with low density of meteorological stations show be analyzed carefully, to avoid introducing some errors into the actual work. The comparison of predicted results under four different emission scenarios may have yielded more comprehensive results, thereby guiding future prevention and quarantine work.

## Supporting Information

S1 File The global distribution of *Rhynchophorus ferrugineus* recorded in CABI. (XLS)

S2 File The known distribution of *Rhynchophorus ferrugineus* in China recorded in references. (XLS)
